# Nup98 recruits the Wdr82–Set1A/COMPASS complex to promoters to regulate H3K4 trimethylation in hematopoietic progenitor cells

**DOI:** 10.1101/gad.306753.117

**Published:** 2017-11-15

**Authors:** Tobias M. Franks, Asako McCloskey, Maxim Nikolaievich Shokhirev, Chris Benner, Annie Rathore, Martin W. Hetzer

**Affiliations:** 1 Laboratory of Molecular and Cellular Biology, Salk Institute for Biological Studies, La Jolla, California 92037, USA;; 2 The Razavi Newman Integrative Genomics and Bioinformatics Core, Salk Institute for Biological Studies, La Jolla, California 92037, USA;; 3 Department of Medicine, University of California at San Diego, La Jolla, California 92093, USA

**Keywords:** Nup98, Wdr82, Set1A, histone 3 Lys4 trimethylation, transcription, acute myeloid leukemia

## Abstract

Recent studies have shown that a subset of nucleoporins (Nups) can detach from the nuclear pore complex and move into the nuclear interior to regulate transcription. One such dynamic Nup, called Nup98, has been implicated in gene activation in healthy cells and has been shown to drive leukemogenesis when mutated in patients with acute myeloid leukemia (AML). Here we show that in hematopoietic cells, Nup98 binds predominantly to transcription start sites to recruit the Wdr82–Set1A/COMPASS (complex of proteins associated with Set1) complex, which is required for deposition of the histone 3 Lys4 trimethyl (H3K4me3)-activating mark. Depletion of Nup98 or Wdr82 abolishes Set1A recruitment to chromatin and subsequently ablates H3K4me3 at adjacent promoters. Furthermore, expression of a Nup98 fusion protein implicated in aggressive AML causes mislocalization of H3K4me3 at abnormal regions and up-regulation of associated genes. Our findings establish a function of Nup98 in hematopoietic gene activation and provide mechanistic insight into which Nup98 leukemic fusion proteins promote AML.

## Abstract (precis)

In this study, Franks et al. investigated the mechanisms underlying how Nup98 regulates gene expression. They show that in hematopoietic cells, Nup98 binds predominantly to transcription start sites to recruit the Wdr82–Set1A/COMPASS complex, which is required for deposition of the histone 3 Lys4 trimethyl (H3K4me3)-activating mark, and expression of a Nup98 fusion protein implicated in aggressive AML causes mislocalization of H3K4me3 at aberrant regions and up-regulation of associated genes due to aberrant Wdr82/Set1A activity.

The nuclear pore complex (NPC) is an ∼125-mDa protein assembly that spans the nuclear envelope (NE) to regulate transport of macromolecules to and from the nucleus of the cell (Wente and Rout 2010; Hoelz et al. 2011; Solmaz et al. 2011; Raices and D'Angelo 2012; Hurt and Beck 2015). The NPC is composed of scaffold nucleoporins (Nups), which form the remarkably stable structure of the nuclear barrel, and peripheral Nups, which decorate the exterior of the NPC to form the cytoplasmic filaments and nuclear basket (Hoelz et al. 2011; Hurt and Beck 2015; Knockenhauer and Schwartz 2016; Beck and Hurt 2017). Traditionally, the NPC was perceived as a static structure whose primary function is to regulate transport at the NE. However, reports suggest that many peripheral Nups are surprisingly dynamic (Rabut et al. 2004), possessing the ability to move off the NPC to affect other nucleoplasmic processes such as mitosis, DNA damage response and repair, and gene expression (Ptak et al. 2014; Ibarra and Hetzer 2015). One such dynamic Nup, Nup98, was first implicated in regulation of nucleoplasmic gene regulation when it was found that its intranuclear dynamics were disrupted by drugs that inhibit RNA polymerase II (Pol II)-mediated transcription (Griffis et al. 2002, 2004). Later, it was determined that Nup98 along with several other peripheral Nups can bind to gene promoters away from the NPC in *Drosophila* and mammalian cells and that depletion of Nup98 protein inhibits transcription at Nup98-bound genes (Capelson et al. 2010; Kalverda and Fornerod 2010; Liang et al. 2013; Franks et al. 2016). While the mechanism of Nup98-mediated gene activation remains unclear, one study showed that Nup98 can interact with the CBP/p300 protein complex (Kasper et al. 1999), an assembly that promotes recruitment of the core transcription machinery and uses its histone acetyltransferase (HAT) activity to promote the formation of open/active chromatin. Furthermore, work in *Drosophila* suggests that Nup98 can recruit the Trx/MLL complex, which promotes histone 3 Lys4 trimethylation (H3K4me3) of a subset of target genes, including the *HOX* gene cluster (Breen and Harte 1993; Petruk et al. 2001; Smith et al. 2011; Pascual-Garcia et al. 2014), and a recent study suggests that MLL1 plays a role in recruiting Nup98 to chromatin in human cells (Xu et al. 2016).

Trx/MLL belongs to a family of Set domain-containing H3K4me3 complexes that is highly conserved across eukaryotes (Schuettengruber et al. 2011; Shilatifard 2006, 2012; Rao and Dou 2015). In yeast, Set1 is the sole enzyme responsible for deposition of the H3K4me3 mark, where it associates with an assembly of accessory factors termed complex of proteins associated with Set1 (COMPASS), which promotes recruitment of Set1 to chromatin and enzyme activation (Lee and Skalnik 2008; Wu et al. 2008; Mohan et al. 2011; Shilatifard 2012; Piunti and Shilatifard 2016). In metazoans, multiple enzyme complexes—including human MLL1/2 (Trx/MLL in *Drosophila*), human MLL3/4 (Trr in *Drosophila*), and Set1A/Set1B (dSet1 in *Drosophila*)—can methylate H3K4; however, each complex differs in the efficiency with which it can deposit H3K4 monomethyl, dimethyl, and trimethyl marks, which have dramatically different effects on expression of adjacent genes (Schuettengruber et al. 2007; Lee and Skalnik 2008; Wu et al. 2008; Shilatifard 2012; Steffen and Ringrose 2014). For example, current paradigms suggest that in mammals, the Set1A/Set1B complex is involved primarily in deposition of H3K4me3, which promotes gene activation at promoters, while MLL1–4 complexes most efficiently deposit H3K4 monomethyl and dimethyl marks, which are important for regulation of enhancer regions located distal to gene promoters (Schuettengruber et al. 2007; Lee and Skalnik 2008). Set1A/Set1B is recruited to chromatin by the COMPASS complex component Wdr82, which binds to RNA Pol II phosphorylated at Ser5 (p-Ser5) (Schuettengruber et al. 2007; Lee and Skalnik 2008). Importantly, it is unknown whether Nup98 plays a role in recruitment of Set1A/B or MLL1–4 complexes to chromatin and, if so, what effect this might have on H3K4 methylation and gene activation.

Understanding how Nup98 regulates expression of target genes remains an extremely relevant question, as many patients suffering from acute myeloid leukemia (AML) harbor mutations in which the N terminus of Nup98 is translocated with the C terminus of one of ∼30 different translocation partners (Xu and Powers 2009; Gough et al. 2011; Franks and Hetzer 2013; Simon and Rout 2014). The mechanism of Nup98 translocation-mediated leukemias is poorly understood. Many of Nup98's C-terminal translocation partners are DNA-binding proteins that, in theory, can bind to chromatin and use the N-terminal Nup98 portion of the translocation to recruit Nup98-binding factors to aberrant chromatin-binding sites (Xu and Powers 2009; Gough et al. 2011; Franks and Hetzer 2013; Simon and Rout 2014). Indeed, the Nup98-Nsd1 fusion, which combines the C-terminal portion of the H3K36me3 methylation enzyme Nsd1 with the N-terminal domain of Nup98, binds to promoters and ORFs of genes that are tightly regulated during hematopoiesis (Wang et al. 2007). As a result, genes such as *Meis1* and *HOXA9*, which are primarily active in hematopoietic progenitor cells (HPCs) and down-regulated upon differentiation, become chronically active, resulting in the inhibition of cell differentiation and the promotion of self-renewal (Wang et al. 2007). This model is challenged by the fact that multiple Nup98 translocations lack DNA-binding domains and thus probably cannot directly disrupt gene expression at aberrant DNA-binding sites (Franks and Hetzer 2013). Interestingly, a recent study demonstrated that MLL1 binds to the N terminus of Nup98 translocation proteins to promote gene activation at developmental genes (Xu et al. 2016). Another study found that the transport factor Crm1, which was shown previously to interact with Nup98, recruits the Nup98-HOXA9 translocation to the *HOX* locus to disrupt gene expression and promote AML (Oka et al. 2016). These findings suggest that the common N-terminal domain and not the C-terminal fusion partner of Nup98 fusion proteins is critical for chromatin recruitment and offers a unifying model for how Nup98 fusions with very different C-terminal translocation partners can trigger similar phenotypes. However, the important question of how recruitment of Nup98 or Nup98 fusion proteins triggers gene activation remains unanswered.

In this study, we aimed to determine how Nup98 activates gene expression in mammalian HPCs in order to understand how Nup98 translocation mutants trigger leukemia. We show that Nup98 binds to intranuclear promoters adjacent to sites associated with H3K4me3 in HPCs with remarkable fidelity. Nup98 interacts and colocalizes with the Set1A/B COMPASS complex component Wdr82 in the nucleoplasm, and loss of Nup98 or Wdr82 leads to inhibition of Set1A recruitment to chromatin and loss of H3K4me3 at promoters. Interestingly, expression of a Nup98 translocation protein (Nup98-Nsd1) leads to atypical deposition of H3K4me3 at sites that colocalize on chromatin with Nup98-Nsd1 binding, offering evidence of Set1A enzyme activity at sites of aberrant Nup98 activity. Our findings describe how Nup98 activates gene expression in healthy cells and offer a new mechanism for how Nup98 translocations disrupt H3K4me3 in HPCs.

## Results

### Nup98 binds to gene promoters adjacent to regions of H3K4me3 in HPCs

**Figure 1.**
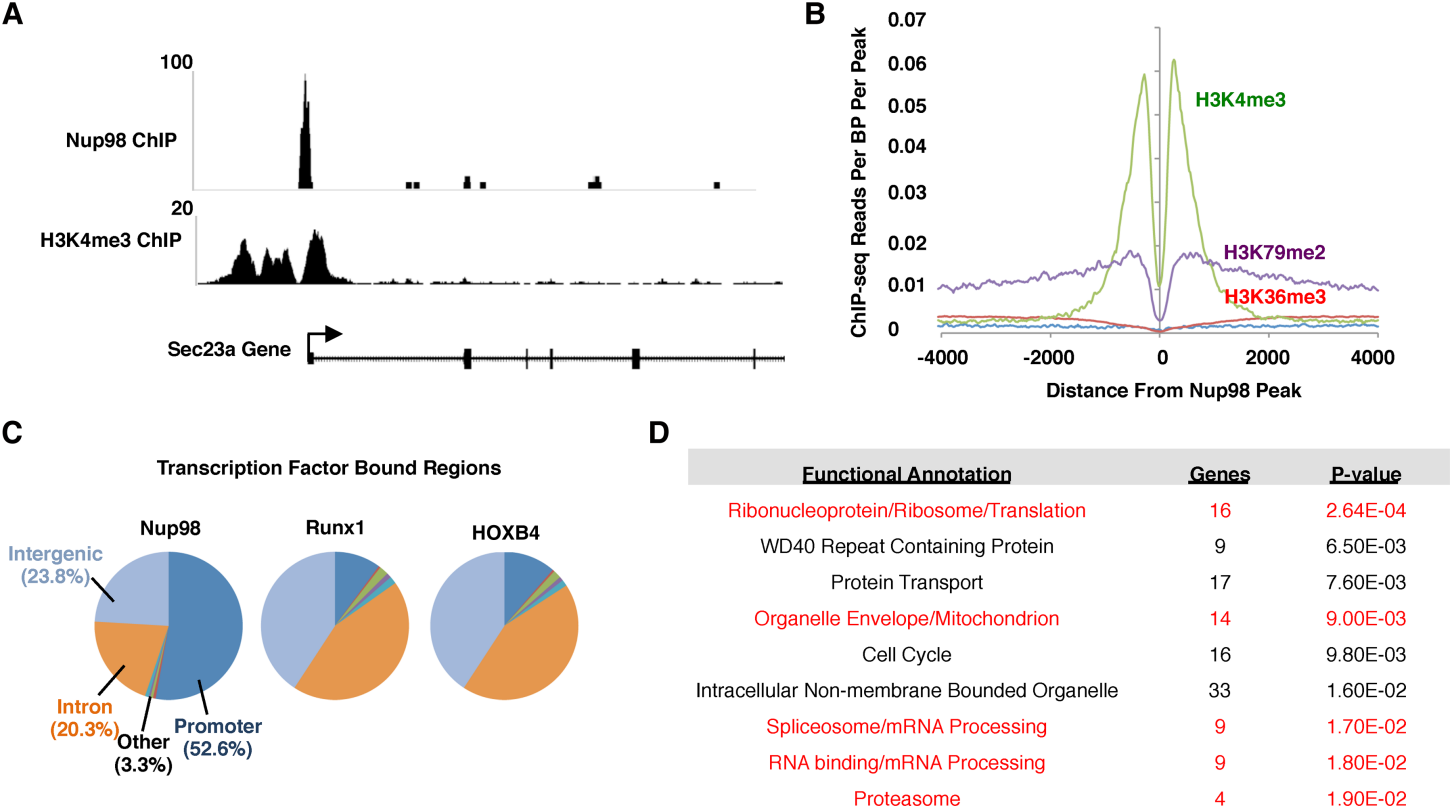
Nup98 binds to transcription start sites adjacent to regions of H3K4me3 in mouse HPCs. (*A*) ChIP-seq trace showing a characteristic peak for Nup98 binding (*top*) and H3K4me3 binding (*bottom*) to an example gene, Sec23a. (*B*) Correlation between H3K4me3 binding and Nup98 binding near transcription start sites. H3K4me3 data were derived from previously published data (Bernt et al. 2011). (*C*) Pie chart showing the percentage of peaks found in a particular region of the genome for Nup98, Runx1 (Wu et al. 2012), and HOXB4 (Fan et al. 2012) ChIP-seq data sets. (*D*) GO analysis results for genes whose promoters were bound by Nup98. Gene clusters and *P*-values were identified using DAVID open access software. Gene clusters that were found to be among the most highly expressed in HPCs are highlighted in red.

As a first step to understand the role of Nup98 in intranuclear gene regulation in HPCs, we wanted to observe how Nup98 interacts with chromatin. To test this, we conducted chromatin immunoprecipitation (ChIP) followed by deep sequencing (ChIP-seq) in mouse HPCs that are immortalized with constitutive *HOXA9* expression (Calvo et al. 2000, 2002). Importantly, these cells still possess the ability to differentiate into most myeloid lineages and thus should provide a relatively accurate representation of how Nup98 functions in wild-type HPCs. We found that most Nup98 ChIP peaks align with gene promoters adjacent to areas of H3K4me3 (Fig. 1A,B; Supplemental Fig. S1A). When we analyzed the preference for Nup98 binding at promoters in comparison with other genomic regions, we found that 52.6% (269 out of 511 peaks) of Nup98 peaks were at promoters (*P*-value < 0.0005) (Fig. 1C). When compared with other transcription factors such as Runx1 and HOXB4, which are known to regulate transcription at promoters in HPCs, Nup98 shows a much greater preference for promoter binding, suggesting a dedicated role in promoter function. Next, we conducted gene ontology (GO) analysis of those promoters that are bound by Nup98. We found that Nup98 interacts primarily with genes involved in “housekeeping” processes such as ribosome biogenesis/translation, protein transport, the cell cycle, splicing, and transcription (Fig. 1D). Notably, all of these gene sets are predicted to be highly expressed in HPCs, and, remarkably, five out of the nine GO terms were also found in our analysis of the most highly expressed gene clusters in HPCs (Supplemental Fig. S1B). We conclude that Nup98 shows a remarkable preference for binding promoters of active genes in HPCs.

### Nup98 interacts with the Set1A/B–COMPASS complex component Wdr82 in the nucleoplasm

**Figure 2.**
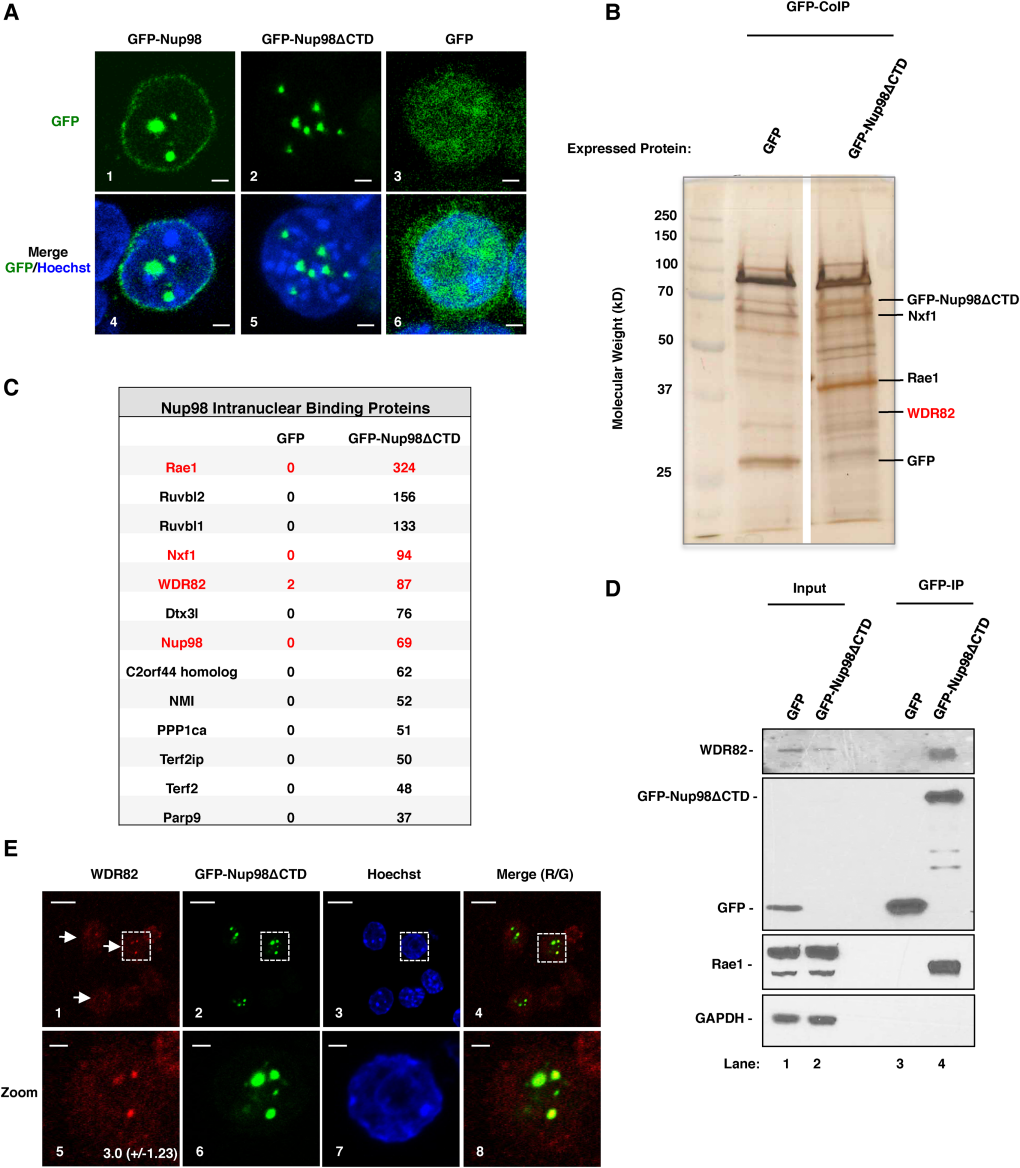
A Nup98 protein variant that localizes only in the nucleoplasm interacts with Wdr82. (*A*) Localization of human GFP-Nup98 (panel *1*), a mutant of Nup98 that does not bind the NPC (GFP-Nup98ΔCTD) (panel *2*), and a control protein (GFP) (panel *3*). GFP-Nup98ΔCTD localizes in GLFG bodies, as has been reported previously (Griffis et al. 2002). Bars, 2 µm. (Panels *4–6*) A merge of GFP and Hoechst staining is shown for each condition. (*B*) Silver-stained sodium dodecyl sulfate (SDS)-PAGE showing proteins that copurified with GFP and GFP-Nup98ΔCTD. Bands corresponding to proteins of interest are indicated at the *right*. (*C*) Table showing most enriched proteins in GFP-Nup98ΔCTD co-IP lysates. Proteins of interest are highlighted in red. The numbers of peptides identified for each protein in control GFP immunoprecipitation and GFP-Nup98ΔCTD immunoprecipitation are indicated. (*D*) Western blots showing input protein and purified protein from GFP-Nup98ΔCTD (lanes *2*,*4*, respectively) and a control protein (GFP) (lanes *1*,*3*, respectively). Input lanes were loaded with 0.625% of starting lysate. (*E*) Immunofluorescence (IF) assays showing localization of Wdr82 (panels *1*,*5*), GFP-Nup98ΔCTD (panels *2*,*6*), Hoechst (panels *3*,*7*), and a merge of Wdr82 and GFP-Nup98ΔCTD localization (panels *4*,*8*). Zoomed images of the dotted box that appears in panels *1–4* are shown in panels *5–8*. The average fold enrichment and standard deviation of Wdr82 focus staining relative to nuclear background are shown in the *bottom right* corner of panel *5*. Bars: panels *1–4*, 10 µm; panels *5–8*, 2 µm.

Our ChIP-seq results suggest that Nup98 might play a role in transcription by modifying promoters. To obtain further insights into the mechanism of Nup98-mediated gene regulation, we wanted to identify proteins that cooperate with Nup98 to regulate transcription. We stably expressed a GFP-tagged human Nup98 mutant (Nup98ΔCTD), which cannot bind the NPC or a negative control protein (GFP) in mouse RAW 264.7 macrophage cells (Fig. 2A, panels 2,3, respectively), and conducted coimmunoprecipitation (co-IP) experiments. Importantly, the inability of GFP-Nup98ΔCTD to bind to NPCs (Fig. 2, panels 2,5) allows us to specifically enrich for those proteins that cooperate with Nup98 to regulate intranuclear transcription. Qualitative evaluation of the coimmunoprecipitated samples revealed that many proteins were enriched in the GFP-Nup98ΔCTD sample as compared with the control GFP sample (Fig. 2B). Indeed, mass spectrometry analysis identified 54 proteins that were significantly enriched in GFP-Nup98ΔCTD immunoprecipitates (Fig. 2C; Supplemental Fig. S2). Nup98 cofactor Rae1, transport factor Nxf1, and Nup98 were among the most enriched Nup98-interacting proteins, suggesting that our immunoprecipitation conditions were highly specific (Fig. 2C). Interestingly, among the most enriched Nup98-interacting proteins, we also identified Wdr82, which promotes H3K4me3 by binding directly to RNA Pol II to recruit the Set1A/B–COMPASS complex to promoters (Lee and Skalnik 2008; Wu et al. 2008). This was exciting considering the observed association of Nup98-binding sites in close proximity to areas of H3K4me3 (Fig. 1A,B). To confirm the interaction between Nup98ΔCTD and Wdr82, we repeated the co-IP experiment and analyzed the purified samples with Western blotting using antibodies against Wdr82, GFP, or Rae1 or a negative control protein, GAPDH (Fig. 2D). Consistent with the mass spectrometry results, Wdr82 and Rae1 were both strongly enriched in the Nup98 co-IP, while GAPDH was not (Fig. 2D, cf. lanes 4 and 3).

If Wdr82 interacts with Nup98ΔCTD, then we predicted that the two proteins should colocalize in intranuclear foci. To test this, we conducted immunofluorescence (IF) experiments with an antibody against Wdr82 in RAW cells stably expressing GFP-Nup98ΔCTD. As shown in Figure 2E and despite the fact that the Wdr82 antibody generally shows only a weak affinity for Wdr82 in IF experiments, we were able to detect distinct colocalization between Wdr82 (panels 1,5) and GFP-Nup98ΔCTD (panels 2,6). We conclude that Nup98 interacts and colocalizes with the Set1A/B–COMPASS complex component Wdr82 in mouse macrophage cells.

### Wdr82 is required for Set1A recruitment to promoters and H3K4me3

**Figure 3.**
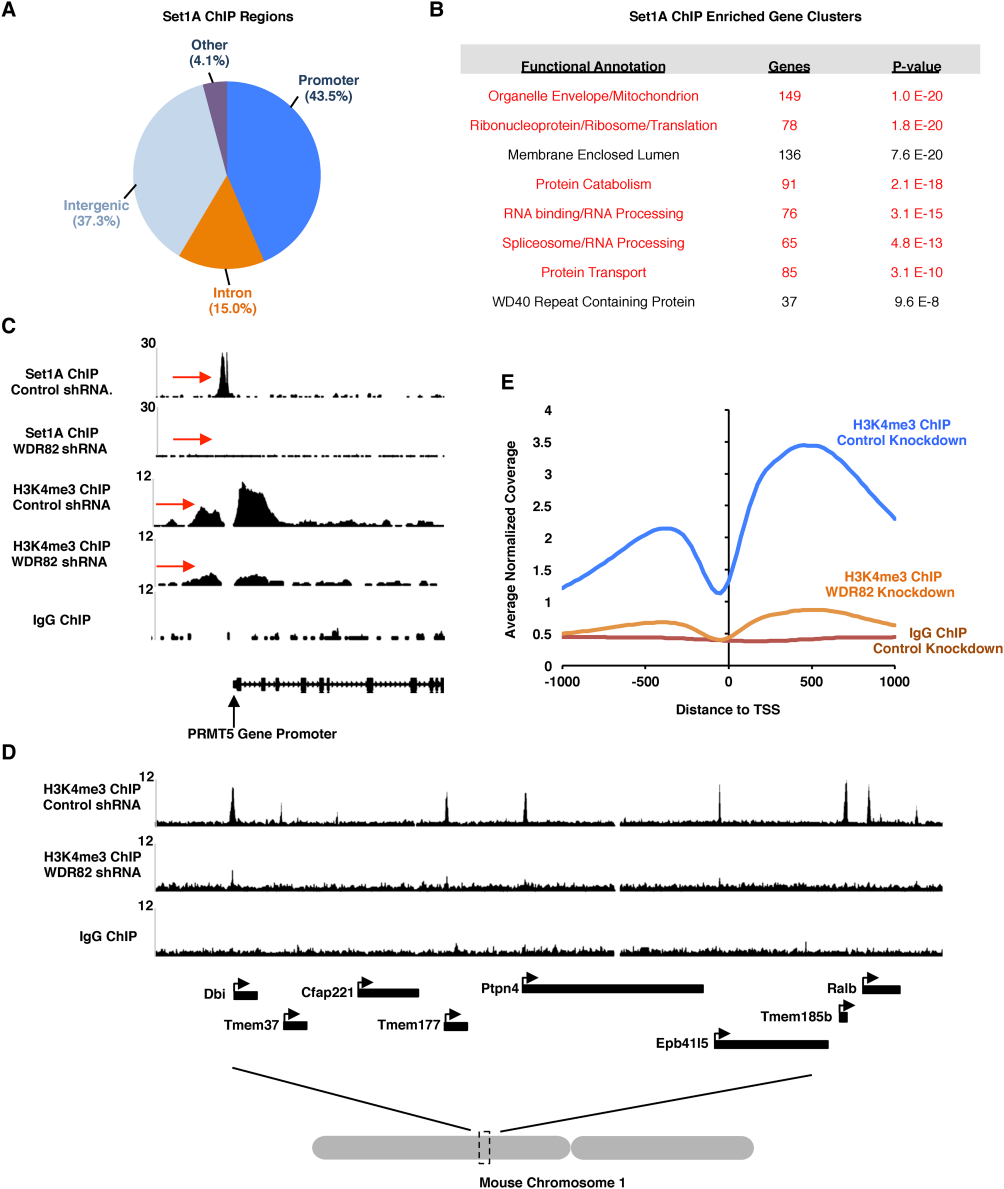
Wdr82 is required for Set1A recruitment to chromatin and H3K4me3. (*A*) Pie chart showing the percentage of Set1A peaks found in different regions of the genome. (*B*) GO analysis of genes whose promoters are bound by Set1A. Groups corresponding to one of the most highly expressed gene clusters in HPCs (Supplemental Fig. S1B) are highlighted in red. (*C*) ChIP-seq traces showing Set1A or H3K4me3 binding to chromatin in control knockdown or Wdr82 knockdown cells. Red arrows indicate regions of interest. (*D*) ChIP-seq traces showing H3K4me3 binding to genomic regions in control knockdown or Wdr82 knockdown cells. (*E*) Genome-wide histogram showing the binding of H3K4me3 in relation to promoter regions for the experimental conditions indicated.

In order to understand the role of Nup98 with Wdr82 in HPCs, we first sought to confirm the function of the Wdr82–Set1A–Compass (WSC) complex in regulating H3K4me3. Recent studies suggest that the WSC complex is the primary entity capable of efficient H3K4me3 in mammals (Lee and Skalnik 2008; Wu et al. 2008). If this were true, then we should be able to detect WSC binding in proximity to transcription start sites and areas of H3K4me3. To test this, we conducted ChIP-seq experiments with antibodies raised against the enzymatic component of the WSC, Set1A, as a Wdr82 antibody suitable for ChIP was not available. We found that, like Nup98, Set1A has a strong preference for promoter binding at sites directly adjacent to areas of H3K4me3 (Fig. 3A,C). In all, 1157 of 2660 Set1a peaks (43.5%) were found to reside within 1 kb of a transcription start site (Fig. 3A). GO analysis of Set1A-binding sites revealed that Set1A, like Nup98, binds the promoters of gene clusters that are ranked among the highest expressing genes in the genome (Fig. 3B; Supplemental Fig. S1B, see those highlighted in red). We next wanted to test whether Wdr82 is required for H3K4me3 in vivo in HPCs. To achieve this, we used a previously described inducible shRNA expression system to efficiently deplete Wdr82 protein levels (Fellmann et al. 2013). We also observed, as was shown previously (Lee and Skalnik 2008; Wu et al. 2008), that Set1A protein levels are also depleted in the absence of Wdr82, indicating that Set1A protein stability is reliant on the presence of Wdr82 (Supplemental Fig. S3A). To determine the effect that Wdr82 knockdown has on Set1A and H3K4me3, we conducted ChIP-seq with antibodies against Set1A or H3K4me3 in control knockdown or Wdr82 knockdown cells. As shown in Figure 3C, Set1A recruitment to chromatin is drastically inhibited in the absence of Wdr82, which correlates with a nearly complete ablation of H3K4me3. This finding suggests that other Set protein complexes (i.e., MLL) cannot compensate in the absence of the WSC complex. Interestingly, we found that H3K4me3 is strongly reduced genome-wide in response to Wdr82 knockdown, not just at those promoters that are bound by Set1A (Fig. 3D,E), suggesting that the WSC is required for H3K4me3 at most if not all active gene promoters. We wondered what effect genome-wide loss of H3K4me3 would have on gene expression. Using RNA sequencing (RNA-seq), we compared expression of Wdr82 knockdown cells with control luciferase (Luc) knockdown cells and found that many genes (3282 genes in total; 1831 genes up-regulated and 1451 genes down-regulated) are significantly misexpressed (adjusted *P*-value < 0.01) upon Wdr82 depletion. Finally, we wished to determine how depletion of H3K4me3 affects the cell growth and viability of HPCs. Taking advantage of the tet-inducible Wdr82 knockdown cell line, we initiated Wdr82 shRNA expression and measured cell growth and viability for 96 h. As shown in Supplemental Figure S3, B–D, cell growth was reduced within 48 h after the induction of Wdr82 knockdown, which correlated with decreased cell viability. We conclude that Wdr82 is essential for Set1A recruitment to gene promoters, genome-wide H3K4me3, and, ultimately, cell survival in HPCs.

### In the absence of Nup98, Set1A recruitment to chromatin is lost, and H3K4me3 is inhibited

**Figure 4.**
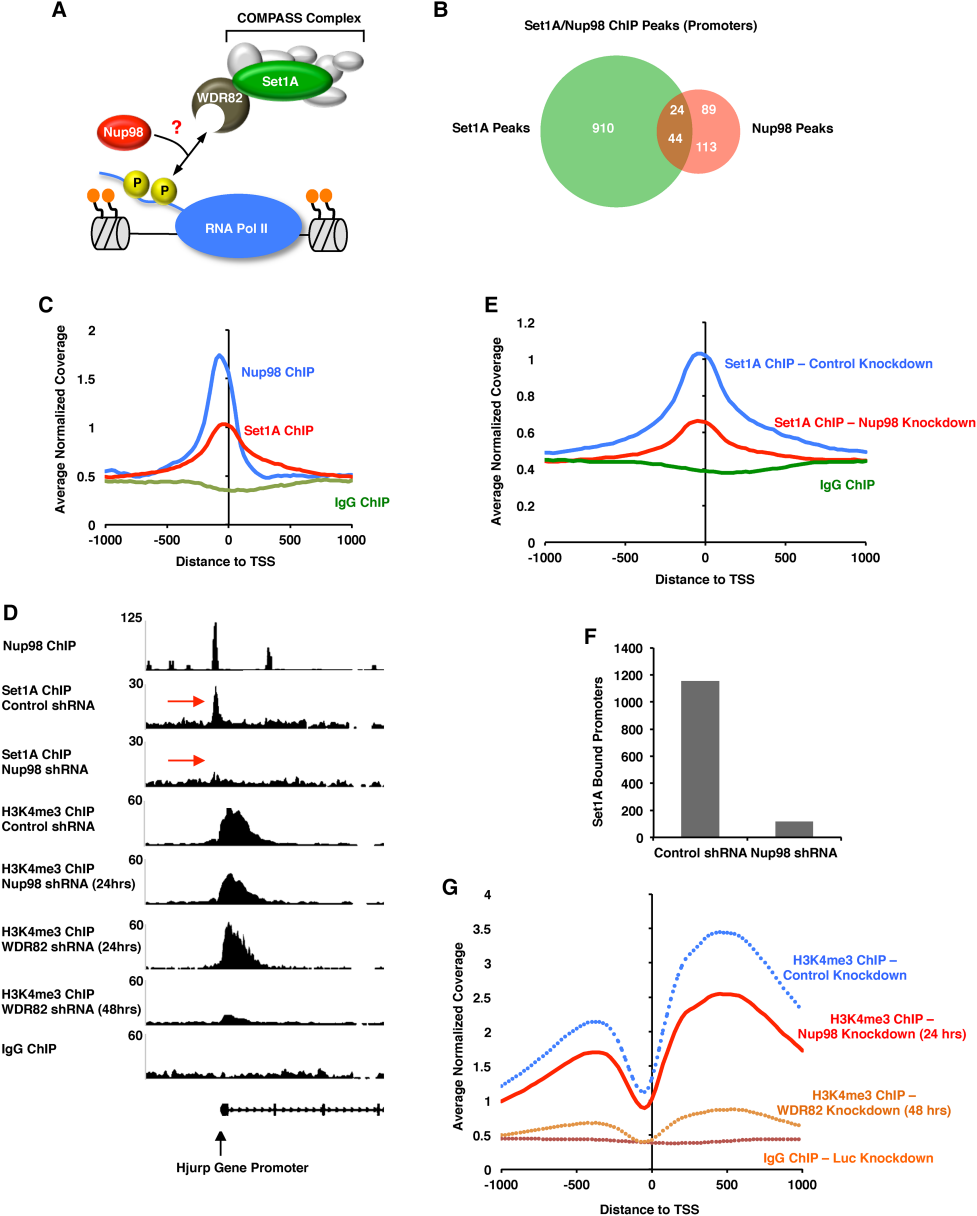
Nup98 is required for Set1A recruitment to chromatin and H3K4me3. (*A*) Model showing the potential role of Nup98–Wdr82 interaction in HPCs. Perhaps Nup98 is required to recruit the WSC complex to chromatin. (*B*) Overlap of Set1A promoter peaks with Nup98 promoter peaks from experiments conducted in mouse HPCs. (*C*) Genome-wide histogram showing the binding of Nup98, Set1A, or a control ChIP (IgG) in relation to promoter regions in wild-type HPCs. (*D*) ChIP-seq trace showing Nup98 binding, Set1A binding in control or Nup98 knockdown conditions, or H3K4me3 binding in control, Nup98 knockdown cells, or Wdr82 knockdown cells in relation to the Hjurp gene promoter. Note that Nup98 knockdown was carried out for 24 h, while Wdr82 knockdown was carried out for 48 h except where indicated in *D*. (*E*) Genome-wide histogram showing the binding of Set1A in relation to promoter regions in control knockdown (blue) or Nup98 knockdown (red) cells. (*F*) Graph showing the number of promoters bound by Set1A in control knockdown or Nup98 knockdown cells. (*G*) Genome-wide histogram showing the binding of H3K4me3 in relation to promoter regions in control knockdown (blue), Nup98 knockdown (red), or Wdr82 knockdown (yellow) or for a control IgG ChIP (brown). Dotted traces indicate data that are the same as shown in Figure 3E.

Since Nup98 interacts with Wdr82 (Fig. 2) and binds chromatin at genomic elements similar to those bound by the WSC component Set1A (Figs. 1, 3), we hypothesized that Nup98 could play a role in recruiting Wdr82 and the rest of the WSC complex to chromatin (Fig. 4A). If this were true, then we would expect a significant overlap between Nup98 ChIP-seq peaks and Set1A ChIP-seq peaks. Indeed, as shown in Figure 4C, both Nup98 and Set1A proteins show a strong preference for binding at promoters in HPCs. Furthermore, when we compared the Nup98 ChIP-seq profiles with the Set1A ChIP-seq profiles, we found that 25.2% of gene promoters bound by Nup98 are also bound by Set1A (*P*-value <0.0005) (Fig. 4B). To test whether Nup98 is required for Set1A recruitment to chromatin, we efficiently depleted Nup98 with shRNAs (Supplemental Fig. S3A) and compared the ChIP-seq profile of Set1A with that obtained from cells treated with a control shRNA. Remarkably, as shown for the example gene in Figure 4D, depletion of Nup98 resulted in a dramatic loss of Set1A recruitment to promoters (red arrows). Moreover, genome-wide analysis revealed that Set1A's strong affinity for binding transcription start sites is lost when Nup98 is depleted (1157 promoter peaks for Set1A in control cells vs. 118 promoter peaks in Nup98 knockdown cells) (Fig. 4E,F). In addition, we were able to observe an ∼30% genome-wide reduction in H3K4me3 at promoters (Fig. 4D,G) despite the fact that we were able to deplete Nup98 for only 24 h due to extreme toxicity to the cells (Supplemental Fig. S4A–C). By comparison, knockdown of Wdr82 did not significantly affect H3K4me3 after 24 h of knockdown (Fig. 4D), as it was too early in the time course to see any change in H3K4me3.

To test whether the effect of Nup98 knockdown on H3K4me3 is more pronounced in cells that tolerate a longer depletion and determine whether the role of Nup98 in H3K4me3 extends beyond mouse HPCs to other cell types and species, we knocked down Nup98 protein expression in human HeLa cells for 72 h and analyzed genome-wide H3K4me3 with ChIP. H3K4me3 was strongly depleted in Nup98 knockdown cells compared with control cells treated with a control siRNA targeting Luc (Supplemental Fig. S4D), suggesting that the role of Nup98 in recruiting the WSC complex to promoters to stimulate H3K4me3 is likely conserved in humans and extends to other cell types besides HPCs.

If Nup98 and Wdr82 are cooperating to regulate many of the same promoters, then it is expected that a gene whose mRNA expression changes (either up or down) in response to Nup98 knockdown should also change in a similar manner when Wdr82 is depleted. Indeed, when we compared those genes significantly misregulated (top 500 most significantly misregulated genes as judged by adjusted *P*-value) by Nup98 knockdown, 73% (364 out of 500) of genes were also misregulated in the same direction in Wdr82 knockdown cells (Supplemental Fig. S4E). This is especially remarkable considering that we were able to deplete Nup98 for only 24 h due to the toxicity of the Nup98 knockdown. We concluded that Nup98 is required for recruitment of Set1A to chromatin, the subsequent deposition of H3K4me3, and proper gene expression regulation.

### The Nup98-Nsd1 translocation stimulates H3K4me3 at ectopic sites to promote aberrant gene activation at developmental genes

**Figure 5.**
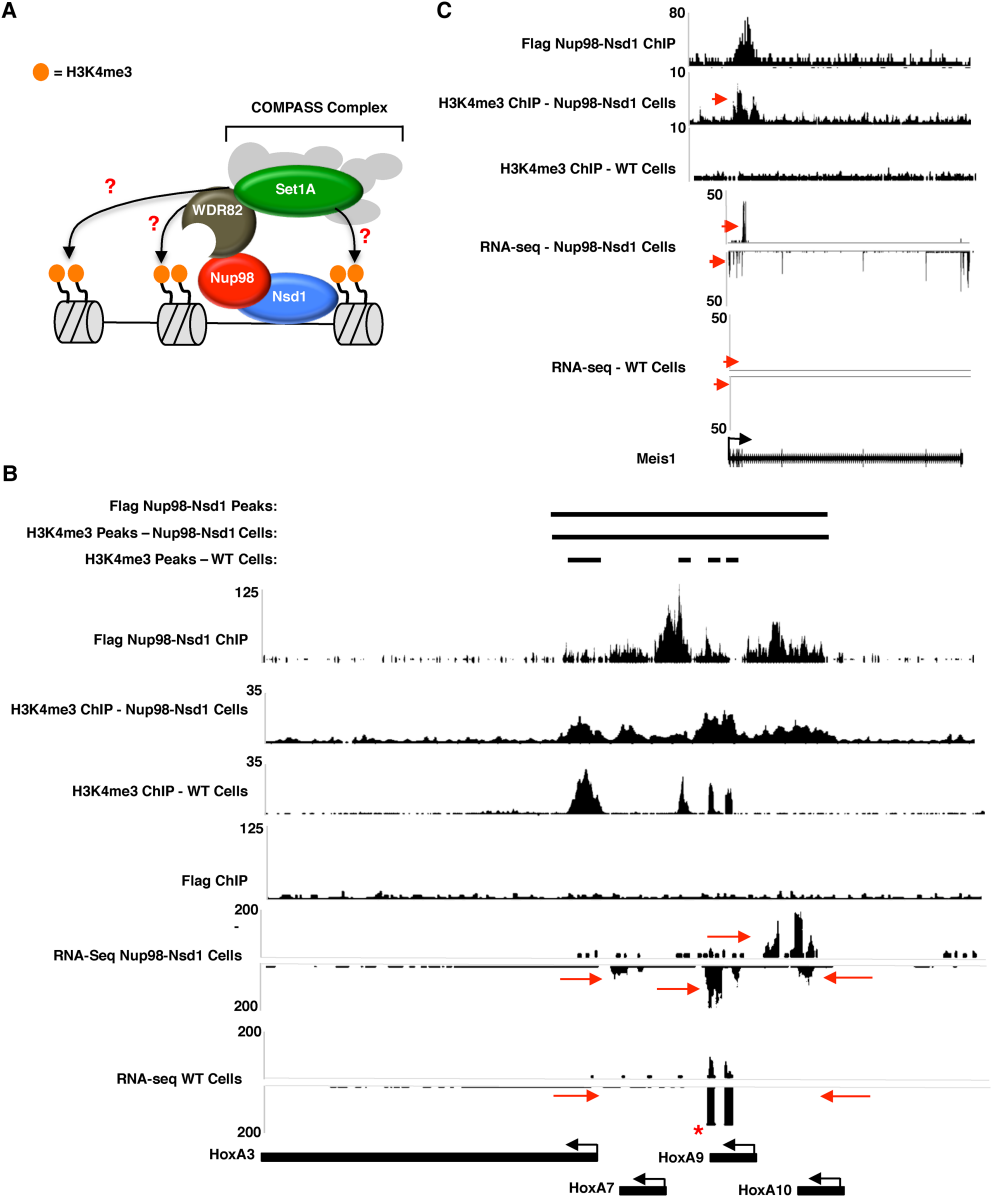
Nup98-Nsd1 expression disrupts H3K4me3 through direct and indirect mechanisms. (*A*) Model showing a possible mechanism by which Nup98-Nsd1 triggers misregulation of genes that it binds. Perhaps Nup98-Nsd1 recruits Wdr82–Set1A to unnatural binding sites to trigger H3K4me3 and gene activation. (*B*,*C*) ChIP-seq traces for Nup98-Nsd1 (top trace) or H3K4me3 in Nup98-Nsd1-expressing (second trace) or wild-type (third trace) cells. RNA-seq tracks for Nup98-Nsd1 or wild-type cells are shown *below* the ChIP-seq tracks. Red arrows indicate regions of interest to compare between wild-type and Nup98-Nsd1 cells. The asterisk indicates exogenous HOXA9 overexpression, which was used to immortalize our wild-type HPC cell line (Calvo et al. 2000).

Previous studies suggest that Nup98 translocation proteins are recruited to the *HOX* locus by MLL1 and/or Crm1, which triggers activation of developmental genes through an unknown mechanism (Oka et al. 2016; Xu et al. 2016). Given that the N-terminal portion of Nup98 is required for the proper recruitment of the WSC complex to chromatin and H3K4me3 in wild-type HPCs (Figs. 2, 4), we predicted that Nup98 translocation proteins promote AML through aberrant recruitment of H3K4me3 activity to developmental genes such as *HOXA* and *HOXB* cluster genes and Meis1 (Fig. 5A). To test this, we conducted ChIP-seq experiments with anti-Flag and anti-H3K4me3 antibodies in mouse HPCs expressing Flag-tagged Nup98-Nsd1 (Wang et al. 2007) and compared the binding profiles at several previously characterized Nup98-Nsd1-binding sites, including a large continuous block that occurs at the *HOX* locus (Wang et al. 2007; Xu et al. 2016). This Nup98-Nsd1-binding site is particularly unusual, as it spans across several intergenic regions, introns, and ORFs between the *HOXA3* promoter and the *HOXA10* ORF (Fig. 5B, top). In wild-type HPCs, H3K4me3 at the *HOX* locus occurs only in well-defined peaks that typically occur around promoter regions (Fig. 5B). In contrast, H3K4me3 at the *HOX* locus in Nup98-Nsd1 cells is spread along the chromatin in a continuous block that resembles the binding pattern of the Nup98-Nsd1 protein (Fig. 5B). This is consistent with our prediction that the Nup98-Nsd1 translocation is able to promote H3K4me3 at unusual genomic sites. We compared the RNA-seq profiles of Nup98-Nsd1 cells and wild-type HPCs at the *HOX* locus and found, as others have shown previously (Wang et al. 2007), that HOX genes falling within the binding region of the Nup98-Nsd1 fusion are strongly up-regulated. These include *HOXA7*, *HOXA9*, *HOXA10*, and an unannotated region that is expressed antisense to the HOXA10 gene (Fig. 5B, red arrows). We observed similar unusual H3K4me3 and up-regulated gene expression at other Nup98-Nsd1-binding sites, including the Meis1 and *HOXB5* loci (Fig. 5C; Supplemental Fig. S5A). Interestingly, the ability of the Nup98-Nsd1 fusion to stimulate unusual H3K4me3 patterns appears to be limited to a subset of binding sites, as we only saw a correlation between Nup98-Nsd1 binding and H3K4me3 at developmental sites that have been previously linked to AML. Thus, it appears that other factors recruited independently at developmental sites help promote the ability of Nup98-Nsd1 to manipulate H3K4me3. We conclude that the Nup98-Nsd1 protein recruits the WSC complex to the *HOXA* and *HOXB* loci and Meis1 gene to promote aberrant H3K4me3, which drives constitutive up-regulation of genes that are normally turned off during hematopoiesis.

## Discussion

### Nup98 is a novel regulator of the WSC complex

**Figure 6.**
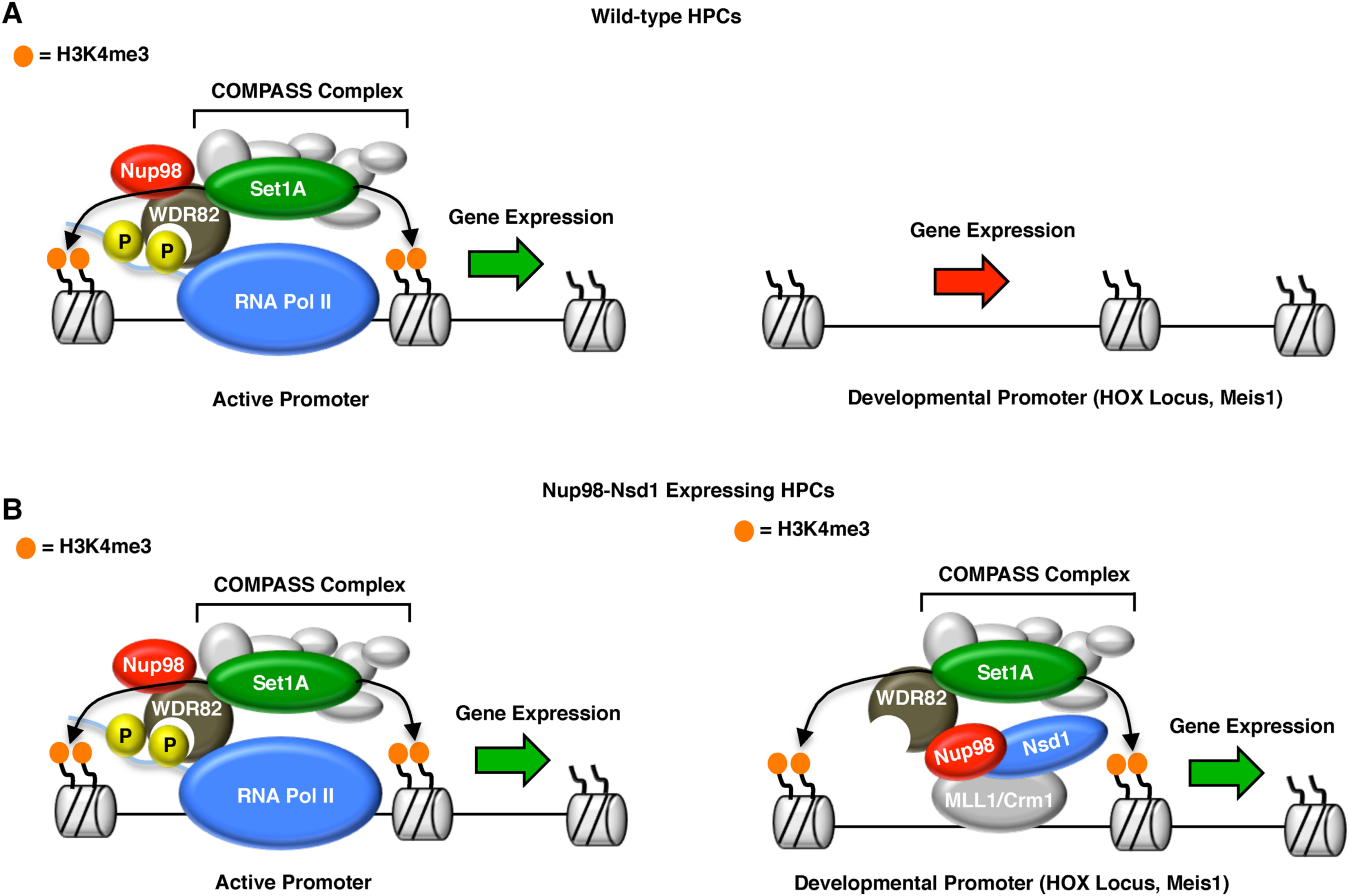
Model for Nup98 function in wild-type and leukemic cells. (*A*) In wild-type cells, Nup98 is required for recruitment of the WSC complex to transcription start sites and thereby promotes H3K4me3 and gene activation. Nup98 is not recruited to promoters of developmental genes, such as those of the HOX locus and Meis1. (*Right* side of diagram) Gene expression at these loci is silent, and cells are poised for differentiation. (*B*) In Nup98-Nsd1-expressing cells, the Nup98 portion of the translocation recruits Set1A activity to the wrong binding sites, which promotes unusual H3K4me3 patterns and constitutive activation of genes that regulate HPC differentiation (shown in the *right* side of diagram).

Here we provide multiple lines of evidence that Nup98 is required for recruitment of the WSC complex to chromatin in HPCs. First, Nup98 interacts and colocalizes with Wdr82 in Nup98 nucleoplasmic foci in mouse macrophage cells (Fig. 2). In addition, both Nup98 and the enzymatic component of the WSC complex, Set1A, preferentially bind to transcription start sites (Figs. 3C, 4C,D) of active genes (Figs. 1D, 3B), and there is a high degree of overlap between Nup98 and Set1A peaks (Fig. 4B,C). Knockdown of Nup98 or Wdr82 inhibits Set1A recruitment to promoters and subsequently leads to a significant reduction of H3K4me3 (Figs. 3E, 4G). Based on these findings, we propose that Nup98 must be a peripheral component of the WSC complex in HPCs (Fig. 6). In the absence of Nup98, Wdr82 is no longer able to bind promoters, recruitment of the WSC complex is lost, and H3K4me3 is ablated as the competition between methylating and demethylating activities shifts in favor of demethylases (Fig. 6). It is still unclear how Nup98 regulates the interaction between Wdr82 and transcription start sites. Previous studies indicate that Wdr82 interacts with chromatin through Ser-5 phosphorylated RNA Pol II (Lee and Skalnik 2008; Wu et al. 2008). We did not detect any RNA Pol II peptides in our Nup98 mass spectrometry samples that would indicate that Nup98 mediates the Wdr82–RNA Pol II interaction (Fig. 2C; Supplemental Fig. S2). In addition, we did not detect an interaction between Nup98 and any other components of the WSC complex, including Set1A. This implies that the Wdr82–Nup98 interaction might stabilize the WSC complex in a way that promotes Wdr82's ability to bind Pol II Ser5 (Fig. 6). Importantly, Set1A is unstable in the absence of Wdr82 but not in Nup98 knockdown cells (Supplemental Fig. S3A). Thus, the Wdr82–Set1A interaction must still be intact in Nup98 knockdown cells, and any change in the complex must be conformational. In the future, we hope to characterize how Nup98 binding affects the WSC complex and its interaction with RNA Pol II. Interestingly, the yeast homolog of Nup98, Nup100, has been implicated in the regulation of transcriptional memory through the maintenance of H3K4 dimethylation (H3K4me2) (Light et al. 2013). Moreover, recent findings suggest that the Set1/COMPASS complex is required for transcriptional memory in yeast (D'Urso et al. 2016). It seems likely that Nup98's regulation of H3K4me2 is somehow related to its role in H3K4me3, but this will be important to characterize in the future, as the roles of Set1 proteins have diversified in humans, with Set1A/COMPASS functioning primarily in H3K4me3, and MLL protein functioning in H3K4me2 and H3K4 monomethylation (Schuettengruber et al. 2007; Lee and Skalnik 2008; Wu et al. 2008; Shilatifard 2012; Steffen and Ringrose 2014).

Notably, recent findings suggest that Nup98 and Nup98 fusion proteins are recruited to chromatin by MLL1 (Pascual-Garcia et al. 2014; Xu et al. 2016). Although our co-IP conditions faithfully capture known Nup98 interaction partners, we did not detect peptides from components of the NSL or MLL1 complexes in our co-IP/mass spectrometry results. Perhaps the interaction between Nup98 and MLL1/NSL proteins is transient, and therefore they are difficult to detect by co-IP. We propose the existence of multiple distinct mechanisms by which Nup98 complexes can be recruited to chromatin in a context-dependent manner. These include recruitment of Nup98 or Nup98 translocations to chromatin through interactions with MLL1 (Xu et al. 2016), Crm1 (Oka et al. 2016), or Wdr82 and the WSC complex as identified in this study. Future studies should explore the potential interdependence or locus specificity of different Nup98 recruitment mechanisms.

### Do all Nup98-mediated leukemias proceed through a common mechanism?

Our results suggest that the Nup98-Nsd1 translocation promotes aberrant gene activation at developmental genes through recruitment of the WSC complex and H3K4me3. We observed that Nup98-Nsd1 binds directly to genes that are important for transcription, cell proliferation, and differentiation (Fig. 5; Supplemental Figs. S5, S6) and promotes their up-regulation through unnatural deposition of the H3K4me3 histone mark (Fig. 6). A key question in the field is whether all Nup98 fusion proteins promote leukemia via the same mechanism or whether the identity of the C-terminal-binding partner determines which genes become misregulated. The fact that many Nup98 fusion-mediated leukemias appear to be triggered by activation of the *HOXA* and *HOXB* loci and Meis1 strongly suggests that the recruitment of the N-terminal portion of Nup98 fusions (through Nup98 itself) is a critical step. In addition, the finding that Nup98 fusions with different translocation partners can be recruited to *HOX* and Meis1 genes (Xu et al. 2016), along with our observation that the N-terminal portion of Nup98 can promote H3K4me3 and gene activation at aberrant sites, provides a unifying model for how Nup98 translocation proteins trigger AML (Fig. 6B). Still, it seems naïve to conclude that the C-terminal translocation partner has no effect on the phenotypes observed in patients suffering from Nup98-mediated leukemias. Notably, different Nup98 fusions trigger slightly different phenotypes in HPCs, ranging from myelodysplastic syndrome to T-cell acute lymphoblastic leukemia to AML (Xu and Powers 2009). The slight variability in phenotype related to different Nup98 translocation partners could be the result of an additional layer of gene expression disruption that is triggered by direct binding of the Nup98 C-terminal translocation partner to genes that are not normally regulated by Nup98. Importantly, several studies have shown that mutation of the DNA-binding domains of Nup98 translocations such as Nup98-Nsd1 and Nup98-*HOXA9* inhibits some cellular leukemic phenotypes (Calvo et al. 2002; Wang et al. 2007). Despite the potential for several different recruitment mechanisms, it seems that once recruited, Nup98 translocation proteins promote gene activation through a common mechanism: recruitment of the WSC complex and aberrant H3K4me3 (Fig. 6B). In the future, we hope to compare H3K4me3 and gene expression in cells from patients expressing different Nup98 translocations to characterize how disruption of H3K4me3 specifically promotes the onset of leukemia in vivo. We hope this will further our understanding of Nup98 fusion-mediated AML and bring the scientific community closer to establishing treatments that can block the progression of this devastating disease.

## Materials and methods

### Cell culture

A description of “wild-type” HOXA9 immortalized mouse HPCs and Nup98-Nsd1 immortalized HPCs can be found in previous publications (Calvo et al. 2000). Wild-type cells were cultured in RPMI 1640 medium (10% FBS, penicillin/streptomycin [Pen Strep], 1:100 dilution of medium harvested from CHO cells that overexpress GMCSF) (Gibco), while Nup98-Nsd1 cells were cultured in Optimem reduced serum medium (10% FBS, 1 × 10^6^ dilution of concentrated β-mercaptoethanol [Fisher], 1:100 dilution of medium harvested from cells that overexpress stem cell factor, Pen Strep) (Gibco). RAW 264.7 cells were cultured in DMEM (10% FBS, Pen Strep) (Gibco). For cell-counting experiments, 250,000 cells were cultured in a 12-well plate, and cells were assayed for viability and live-cell number at the indicated time points. For shRNA knockdown of Wdr82, Nup98, or Luc control, shRNA oligo sequences (see the Supplemental Material) were cloned into the previously characterized all-in-one inducible knockdown system (Fellmann et al. 2013). Vectors were used to produce lentivirus in 293T cells that was used to infect wild-type HOXA9 immortalized HPCs. Cells were selected for >7 d in puromycin. Doxycycline was added to cells at a final concentration of 1 µg/mL to induce shRNA expression.

### ChIP-seq

Cells (40 × 10^6^) were fixed in 1% paraformaldehyde (PFA) for 10 min, and ChIP-seq was performed as described previously (Liang et al. 2013; Jacinto et al. 2015). The following antibodies were used for ChIP: Nup98 (purchased from Cell Signaling Technologies, P671, no.2292), H3K4me3 (purchased from Abcam, ab8580), Set1A (purchased from Abcam, ab70378), and Flag (purchased from Sigma, Flag M2 affinity gel, no. A2220). Reads were aligned to the mouse genome (mm10 and GRCm38) (Figs. 1–5) or human genome (hg19 and GRCh37) (Supplemental Fig. S4D) using bwa (version 0.7.12) (Li and Durbin 2009). Only reads that aligned uniquely to a single genomic location (MAPQ > 10) were used for downstream analysis. ChIP-seq peaks and normalized bedGraph files were generated using HOMER using a false discovery rate of 0.1% and fold enrichment over input of at least fourfold (Heinz et al. 2010). Data used to characterize binding of genomic elements by RUNX1 and HOXB4 (Fig. 1C) were obtained through Gene Expression Omnibus from Fan et al. (2012) and Wu et al. (2012).

### RNA-seq analysis

Following doxycycline-induced knockdown of Nup98 (24 h), Wdr82 (48 h), or a control protein (Luc; 24 or 48 h depending on experiment), 5 × 10^6^ cells were washed with 1× PBS, and RNA was isolated using the RNeasy (Qiagen) purification kit. Libraries were prepared using the Illumina RNA library preparation kit. Reads were aligned to the mouse genome (mm10 and GRCm38) using STAR (version 2.2.0.c) (Dobin et al. 2013). Only reads that aligned uniquely to a single genomic location were used for downstream analysis (MAPQ > 10). Gene expression values were calculated for annotated RefSeq genes using HOMER by counting reads found overlapping exons (Heinz et al. 2010). Differentially expressed genes were found using EdgeR (Robinson et al. 2010). GO functional enrichment analysis was performed using DAVID (Dennis et al. 2003).

### Co-IP mass spectrometry analysis of Nup98-interacting proteins

GFP-tagged human Nup98ΔCTD (amino acids 1–504) or, as a control, GFP alone was cloned in the PQCXIB retroviral expression vector. Retrovirus was produced in 293T cells and used to infect RAW 264.7 mouse macrophage cells. After 7–14 d of blasticidin selection, cells were sorted for positive GFP expression. Cells were allowed to expand, and 50 × 10^6^ cells for each cell type (GFP or GFP-Nup98ΔCTD) were harvested by cell scraping. Cells were spun at 1500*g* and washed with 1× PBS. Cells were lysed for 10 min on ice in 2 mL of co-IP lysis buffer (0.1% Triton X-100, 0.25% sodium deoxycholate, 150 mM NaCL, 1 mM EDTA, 10 mM Tris at pH 7.5) and spun at 1500*g* to pellet insoluble debris. Lysate was removed and placed in a separate tube containing 50 µL of GFP-Trap metallic beads (Bulldog Bio) that had been preblocked for >1 h in immunoprecipitation blocking buffer (0.5% BSA in 1× PBS). After 2–4 h of incubation of protein lysate with GFP-TRAP beads, the lysate was aspirated, and beads were washed four times with Net-2 buffer (50 mm Tris at pH 7.5, 50 mM NaCL, 1 mM EDTA, 0.1% Triton-X 100). Protein was eluted from the beads by adding 50 µL of 2× sodium dodecyl sulfate (SDS) load buffer and incubating for 5 min at 95°C. Twenty-five microliters of lysate was run on SDS-PAGE and stained with Simple Blue stain (Invitrogen), and gel pieces were cut and subjected to mass spectrometry analysis. Those proteins with more than fivefold enrichment of peptide identification in the GFP-Nup98ΔCTD condition over the GFP control are listed in Supplemental Figure S2.

### IF assays

RAW 264.7 macrophage cells grown on cover slips in 24-well plates were washed once with 1× PBS and fixed for 5 min in 4% PFA. After three washes with PBS, cells were incubated for 10 min in 1× IF buffer (10 mg/mL BSA, 0.1% Triton X-100, 0.02% SDS, 1× PBS). Next, cells were incubated for 2 h in IF buffer containing an antibody against rabbit WDR82 (a gift from David Skalnik) at 1:100 dilution (Fig. 2). Cells were washed three times and incubated in 1:2000 dilution of anti-rabbit Alexa 568 secondary antibody (Life Technologies) for 1 h. After five washes, cells were incubated with 1 µg/mL Hoechst DNA stain for 5 min. Cells were washed one additional time and mounted on slides.

## Supplementary Material

Supplemental Material
