# Supplementary material for: Nup98 recruits the Wdr82–Set1A/COMPASS complex to promoters to regulate H3K4 trimethylation in hematopoietic progenitor cells
Source: Genes Dev. 2017 Nov 15;31(22):2222–34. doi: 10.1101/gad.306753.117 (PMC5769767; doi:10.1101/gad.306753.117)
Supplement: Supplemental Material [file supp_31_22_2222__index.html]

Nup98 recruits the Wdr82–Set1A/COMPASS complex to promoters to regulate H3K4 trimethylation in hematopoietic progenitor cells — Supplemental Material 

# Nup98 recruits the Wdr82–Set1A/COMPASS complex to promoters to regulate H3K4 trimethylation in hematopoietic progenitor cells

## Supplemental Material

- Supplemental\_Material.pdf
