# Supplementary material for: Nup98 recruits the Wdr82–Set1A/COMPASS complex to promoters to regulate H3K4 trimethylation in hematopoietic progenitor cells
Source: Genes Dev. 2017 Nov 15;31(22):2222–34. doi: 10.1101/gad.306753.117 (PMC5769767; doi:10.1101/gad.306753.117)

Supplemental Material

Oligonucleotides used to clone miRE knockdown vectors

MiRE-Luc-F ("Control shRNA")
TGCTGTTGACAGTGAGCGCAGGAATTATAATGCTTATCTATAGTGAAGCCACAGATG
TATAGATAAGCATTATAATTCCTATGCCTACTGCCTCGGA

MiRE-Luc-R
TCCGAGGCAGTAGGCATAGGAATTATAATGCTTATCTATACATCTGTGGCTTCACTA
TAGATAAGCATTATAATTCCTGCGCTCACTGTCAACAGCA

MiRE-mWDR82-F
TGCTGTTGACAGTGAGCGCCAGTCGTTTACAGCTCTAACATAGTGAAGCCACAGAT
GTATGTTAGAGCTGTAAACGACTGTTGCCTACTGCCTCGGA

MiRE-mWDR82-R
TCCGAGGCAGTAGGCAACAGTCGTTTACAGCTCTAACATACATCTGTGGCTTCACTA
TGTTAGAGCTGTAAACGACTGGCGCTCACTGTCAACAGCA

MiRE-mNup98-F
TGCTGTTGACAGTGAGCGACACAACCAGTGCTCCTTCATATAGTGAAGCCACAGAT
GTATATGAAGGAGCACTGGTTGTGCTGCCTACTGCCTCGGA

MiRE-mNup98-R
TCCGAGGCAGTAGGCAGCACAACCAGTGCTCCTTCATATACATCTGTGGCTTCACTA
TATGAAGGAGCACTGGTTGTGTCGCTCACTGTCAACAGCA

Supplemental Figure Legends

Supplemental Figure 1. Nup98 binds to chromatin in the vicinity of H3K4me3 regions in mouse HPCs. (A) Nup98 and H3K4me3 ChIP-seq traces on chromosome 8 in mouse HPCs. A zoomed region indicated by the dotted box is enlarged at the bottom. Nup98 and H3K4me3 called peaks are indicated by vertical black tick marks. (B) Graph showing the highest expressed gene clusters determined by analyzing the 1000 most expressed genes in mouse HPCs using DAVID. Gene clusters are ordered by p-value. (C) DNA adenine methyltransferase identification (DAM-ID) fusion proteins used to

conduct DAM-ID to characterize binding of wt Nup98 and Nup98ΔCTD in mouse HPCs. (D) Genome browser region selected to show significant overlap between wt Nup98 and Nup98ΔCTD in mouse HPCs. (E) Venn diagram showing overlap of wt Nup98 and Nup98ΔCTD DAMID peaks. (F) Venn diagram showing overlap of wt Nup98 and Nup98ΔCTD bound promoters.

Supplemental Figure 2. Complete results from Co-IP/Mass-spec experiment in mouse RAW macrophage cells. Mouse Raw macrophage cells stably expressing a control protein GFP or GFP-Nup98ΔCTD were purified using Co-IP. Purified proteins were subjected to SDS-PAGE and bands were cut and analyzed by mass spec. The number of peptides for each protein identified in the GFP-Nup98ΔCTD sample are shown on the right, while the number of peptides that purified with the control protein GFP are shown in the left column.

Supplemental Figure 3. WDR82 knockdown causes slow growth and cell death in mouse HPCs. (A) (Left side) Western blot showing protein levels for Nup98, Nup96, Set1A or a control protein GAPDH in the presence of a control shRNA targeting luciferase, shNup98 or shWDR82. (Right side) Western blot showing protein levels of WDR82, Set1A, MLL1 and Tubulin in WDR82 knockdown cells. Set1a is depleted in the presence of Wdr82 knockdown while MLL1 is not. (B) Qualitative observation of cell growth and viability in cells treated with shWDR82. Brightfield images are shown in panels 1-5 while GFP (indicating activation of shRNA expression in response to DOX) images are shown in panels 6-10. The percentage of cells alive is indicated in the bottom right corner of each GFP image. See Figure S4A to see control cells whose cell growth and viability were used to compare with shWDR82 knockdown cells. (C-D) Graphs

comparing % cells alive and total cell number over a time course for cells treated with a control shRNA targeting luciferase or an shRNA targeting WDR82.

Supplemental Figure 4. Nup98 knockdown is lethal for mouse HPCs. (A) Qualitative observation of cell growth and viability in cells treated with shNup98. Brightfield images for control and Nup98 knockdown cells are shown in panels 1-5 and 11-15, respectively while GFP (indicating activation of shRNA expression in response to DOX) images for control and Nup98 knockdown cells are shown in panels 6-10 and 16-20, respectively. The percentage of cells alive is indicated in the bottom right corner of each GFP image. (B-C) Graphs comparing % cells alive and total cell number over a time course during which cells were treated with a control shRNA targeting luciferase or shNup98. (D) Genome wide histogram showing the relative binding of H3K4me3 in relation to promoter regions in control and Nup98 knockdown cells. (E) Graph comparing expression of the top 500 most misregulated genes in Nup98 knockdown cells (as determined by adjusted p-value) with expression of the same genes in Wdr82 knockdown cells. Each red dot represents a gene. Those red dots falling within the grey boxes in the upper right and lower left quadrant of the graph represent genes changing in the same direction (up or down, respectively) in response to either Nup98 or Wdr82 depletion.

Supplemental Figure 5. Unusual H3K4me3 at HOXB locus overlapping with Nup98-Nsd1 binding. (A) ChIP-seq trace showing interaction of the indicated protein with the HOXB locus. Peaks called for each ChIP are shown at top of the figure. (B) Charts showing gene ontology analysis for the 1000 genes most misregulated in Nup98-Nsd1 expressing cells as compared to wild-type HOXA9 immortalized HPCs.

Supplemental Figure 6. Nup98-Nsd1 binds chromatin and triggers gene misregulation in mouse HPCs. (A) Schematic of the Flag-Nup98-Nsd1 protein as compared to wild-type Nup98 and a Nup98 mutant that can only localize in the nucleoplasm, Nup98-ΔCTD. Note that the portion of Nup98 that is fused with Nsd1 to make the Nup98-Nsd1 fusion protein is very similar to the Nup98-ΔCTD protein that was used to identify the interaction between Nup98 and the WDR82-Set1A-COMPASS complex (Figure 2). (B) Summary of genomic elements bound by by Nup98-Nsd1 protein in mouse HPCs. (C) Gene ontology analysis of genes enriched for Nup98-Nsd1 binding in mouse HPCs. (D) Heat map showing dramatic mRNA misregulation between Nup98-Nsd1 expressing HPCs and wild-type HPCs. (E) Graph comparing gene expression in Nup98-Nsd1 cells relative to wild-type cells. Genes misexpressed > log2 of 2-fold are highlighted in red. Meis1 and HOX genes discussed elsewhere in the manuscript are highlighted. The line of dots (genes) that appears in the graph is caused by the fact that we had many genes whose fpkm values were "0" for wild-type cells, but much higher for Nup98-Nsd1 expressing cells. Meis1 is an example of one of these genes. Unfortunately, we cannot calculate log2 ratios when "0" values are in the calculation and thus genes like Meis1 would be left out of the data set. To circumvent this, we gave these genes in wild-type cells an arbitrary fpkm of 1, which causes both the total FPKM and FPKM ratio (Nup98-Nsd1/Wild-type expression) values to equal each other. This allows us to visualize the expression of relevant genes like Meis1 that would otherwise be binned out of the analysis. (F) Venn diagram showing overlap of Nup98-Nsd1 peaks with genes misregulated in Nup98-Nsd1 expressing cells.

Lee, J.H., and Skalnik, D.G. (2008). Wdr82 is a C-terminal domain-binding protein that recruits the Setd1A Histone H3-Lys4 methyltransferase complex to transcription start sites of transcribed human genes. Molecular and cellular biology *28*, 609-618.

Wu, M., Wang, P.F., Lee, J.S., Martin-Brown, S., Florens, L., Washburn, M., and Shilatifard, A. (2008). Molecular regulation of H3K4 trimethylation by Wdr82, a component of human Set1/COMPASS. Molecular and cellular biology *28*, 7337-7344.

A

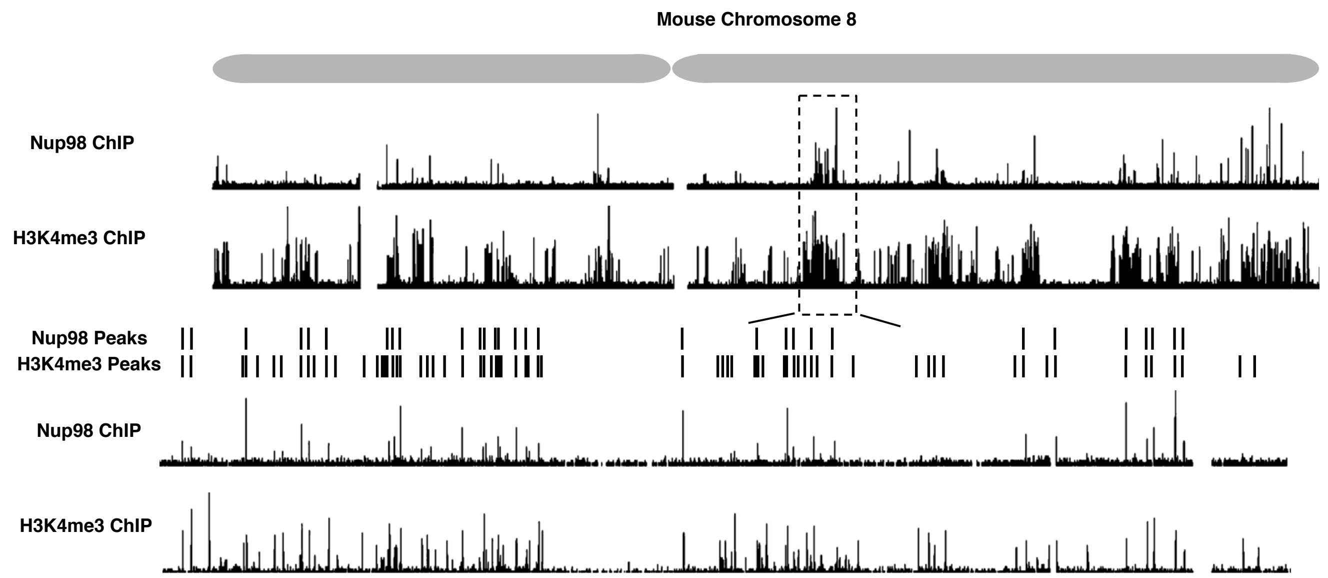

B

HPCs – Highest Expressed Gene Clusters

Functional Annotation	Genes	P-value
Ribonucleoprotein/Ribosome/Translation	101	1.80E-53
Mitochondrion/Electron Transport	148	3.30E-38
RNA binding/mRNA Processing	129	3.00E-33
Cell-Cell Adhesion	70	6.60E-29
Protein Folding/Chaperone	48	3.10E-27
Proteasome	31	1.20E-26
mRNA Splicing	55	2.60E-24
Protein Biosynthesis	41	1.40E-21
Endoplasmic Reticulum	135	2.20E-16
Citric Acid Cycle	20	2.00E-13

C

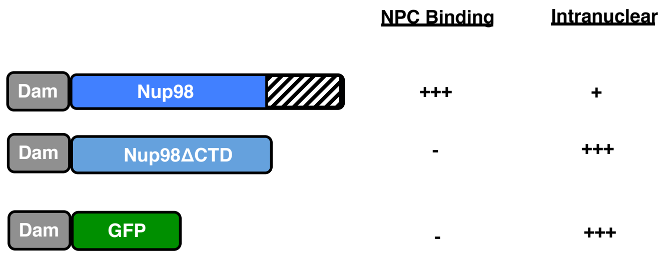

D

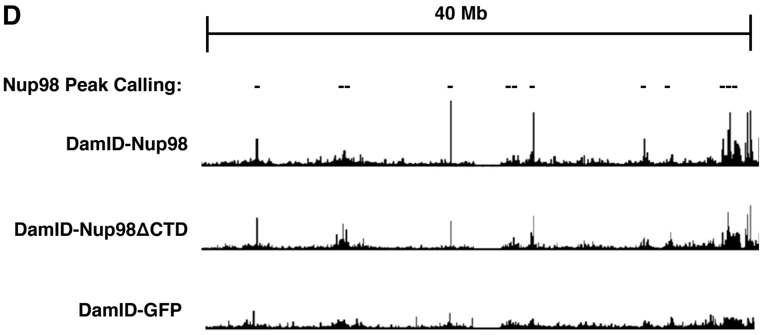

E

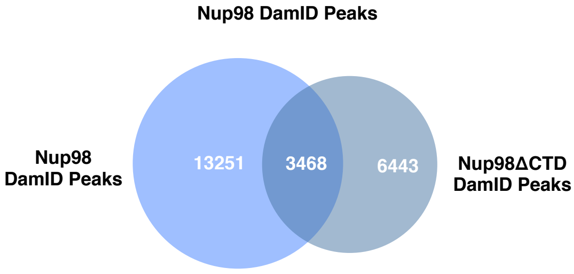

F

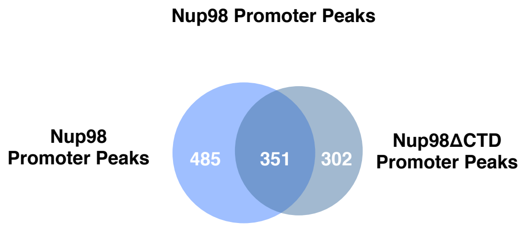

Nup98 Intranuclear Binding Proteins - RAW cells		
	GFP	GFP-Nup98ΔCTD
Rae1	0	324
Ruvbl2	0	156
Ruvbl1	0	133
Nxf1	0	94
WDR82	2	87
Dtx3l	0	76
Nup98	0	69
C2orf44 homolog	0	62
NMI	0	52
PPP1ca	0	51
Terf2ip	0	50
Terf2	0	48
Parp9	0	37
Zc3h4	0	36
Nupl2	0	25
Prpf8	1	24
Tox4	1	22
Rpl7	2	20
PPP1cb	1	18
Rplp2	2	16
Rpl4	0	14
Zfml	0	14
Elavl1	1	12
Rpl3	2	12
Itgb4	0	12
Gm6636	2	10
Cpsf1	2	10
Tfg	2	10
Rps16	0	10
Pcf11	2	10
P4hb	0	8
Lgals3	0	8
Cltc	0	8
Eno1	0	8
Rpl13a	0	7
Rps9	0	7
Rpl9	1	7
Capg	0	7
Rps18	0	7
Rps4x	1	6
Anxa5	0	6
Rps3a	0	6
Gm11353	0	6
Fbl	0	6
Raly	0	6
Rpl23a	0	6
Rpl17	0	5
Cpsf2	0	5
ATP5b	0	5
60S - L18like	0	5
GM6316	0	5
Chtf8	0	5
GM5611	0	5
DDX21	0	5

Franks et al._Figure S3

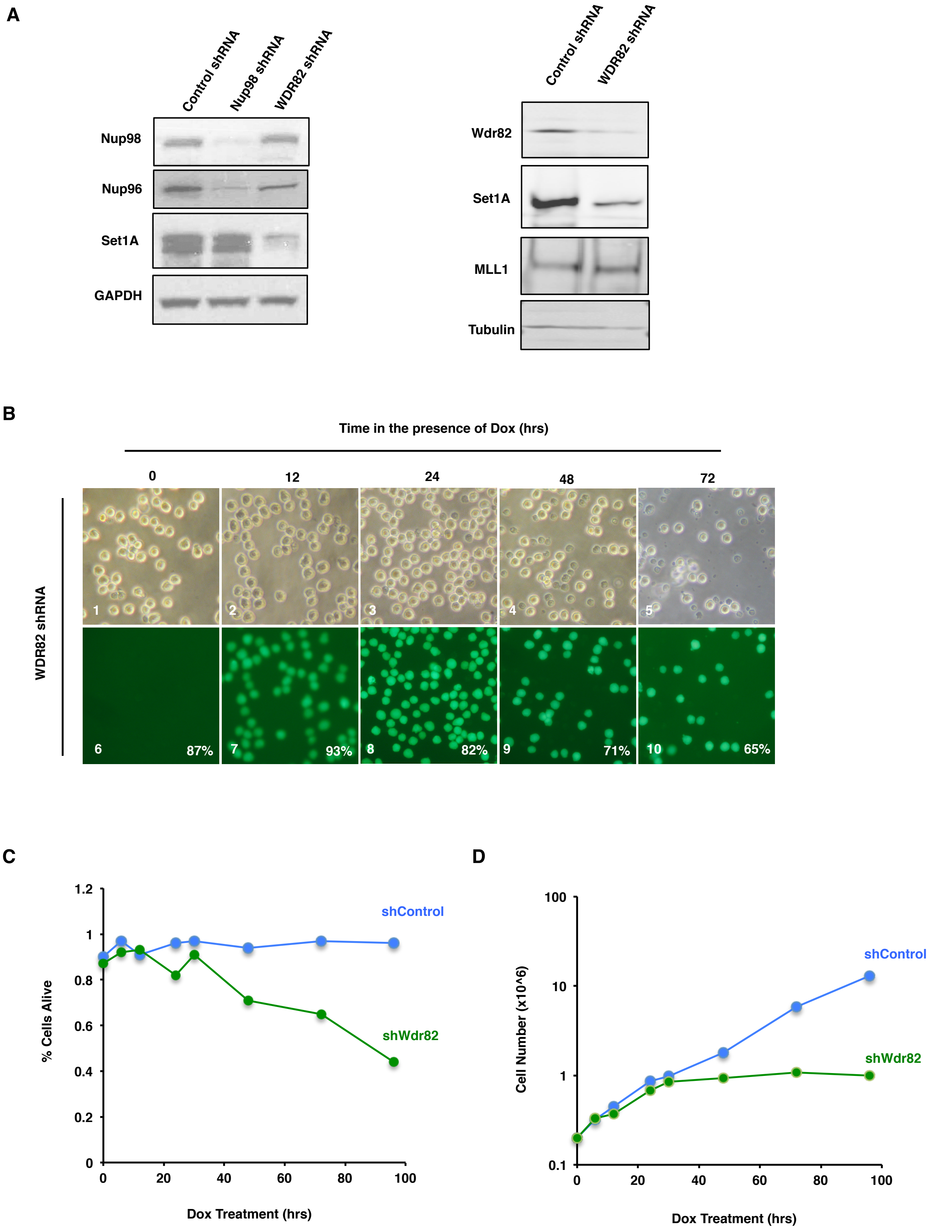

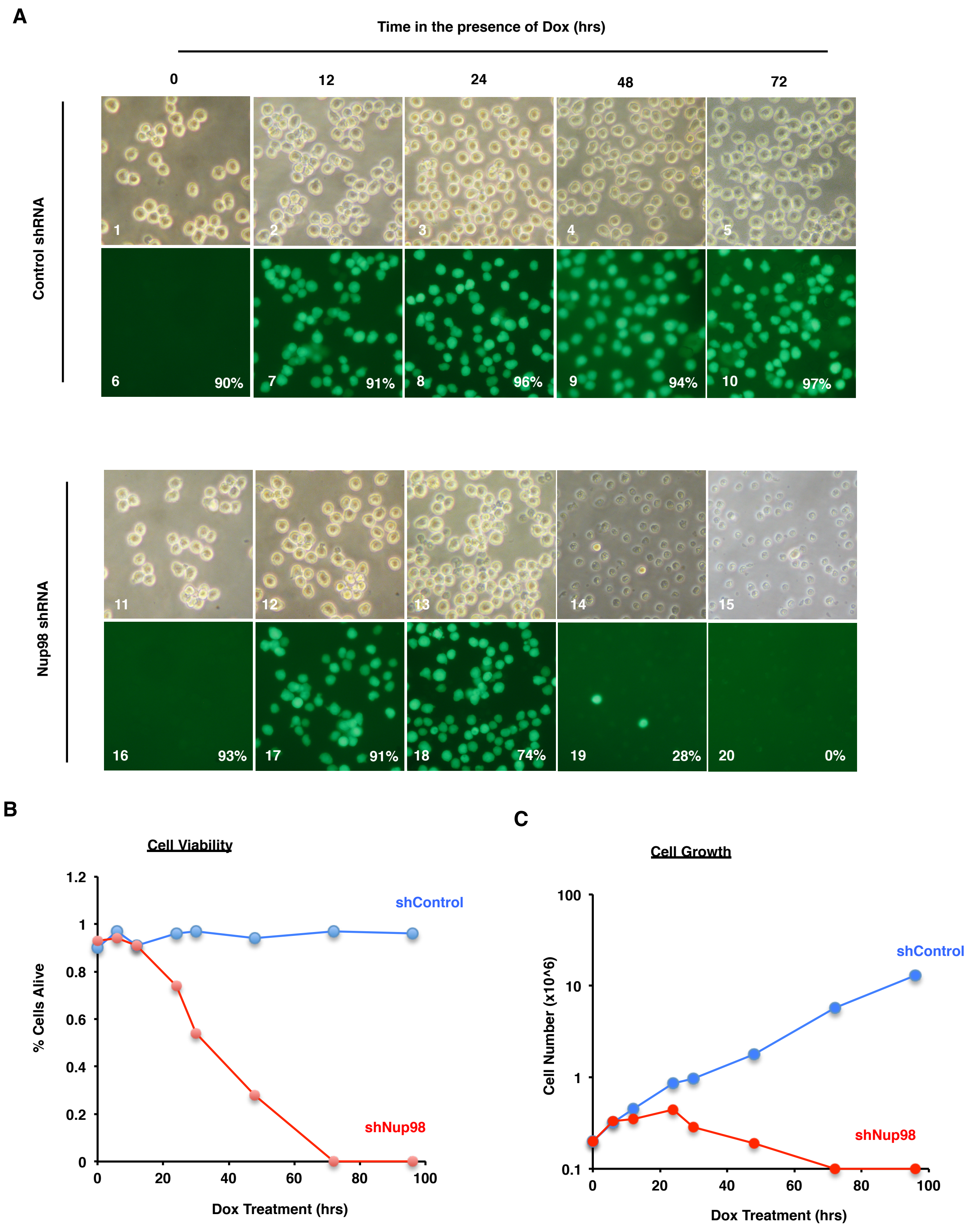
A
Time in the presence of Dox (hrs)
0
12
24
48
72
Control shRNA
1
2
3
4
5
6
90%
7
91%
8
96%
9
94%
10
97%
Nup98 shRNA
11
12
13
14
15
16
93%
17
91%
18
74%
19
28%
20
0%
B
Cell Viability
% Cells Alive
1.2
1
0.8
0.6
0.4
0.2
0
0
20
40
60
80
100
Dox Treatment (hrs)
shControl
shNup98
C
Cell Growth
Cell Number (x10^6)
100
10
1
0.1
0
20
40
60
80
100
Dox Treatment (hrs)
shControl
shNup98

D

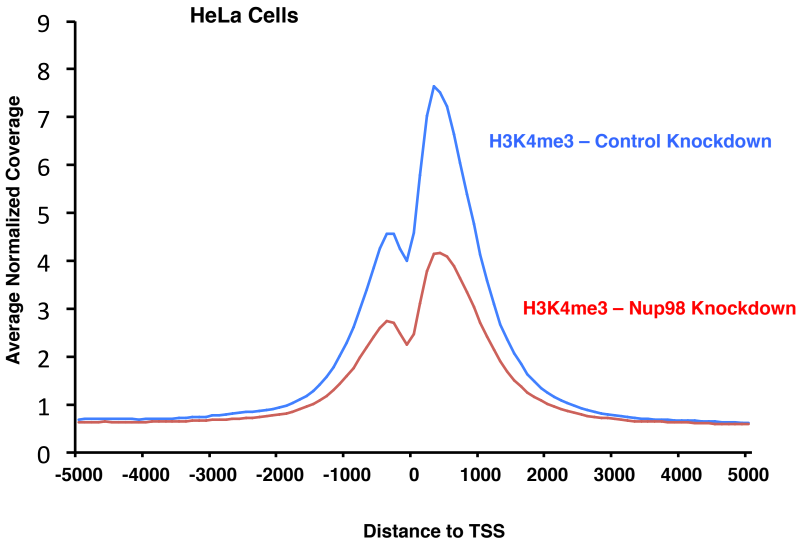
HeLa Cells
Average Normalized Coverage
9
8
7
6
5
4
3
2
1
0
H3K4me3 – Control Knockdown
H3K4me3 – Nup98 Knockdown
-5000
-4000
-3000
-2000
-1000
0
1000
2000
3000
4000
5000
Distance to TSS

E

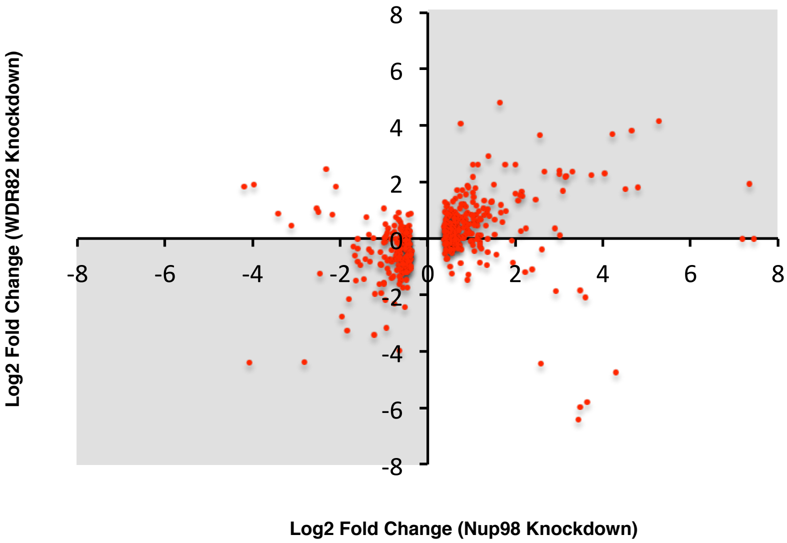
Log2 Fold Change (WDR82 Knockdown)
8
6
4
2
0
-2
-4
-6
-8
-8
-6
-4
-2
0
2
4
6
8
Log2 Fold Change (Nup98 Knockdown)

A

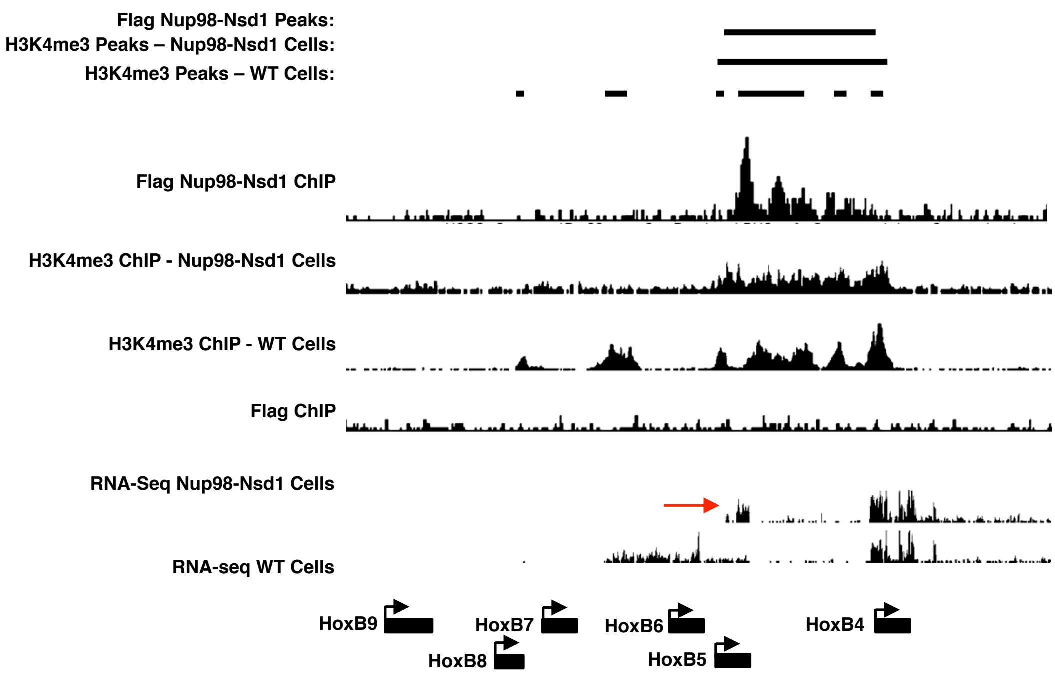

B

Nup98-Nsd1 RNA-Seq (Genes Down)

Functional Annotation	Genes	P-value
Supressor of Cytokine Signaling	4	1.4 E-05
Extracellular Protein	29	5.9 E-05
Membrane Protein	118	1.3 E-04
Intracellular Signaling Cascade	31	1.6 E-04
GTP Binding	16	3.0 E-04
Guanyl Nucleotide Binding	14	4.0 E-04
Glycoprotein	81	7.0 E-04
Disulfide Bond Containing	60	7.8 E-04
Lipoprotein	21	1.2 E-03
Transmembrane Receptor (Serine/ Threonine Kinase Sig.)	7	1.6 E-03

Nup98-Nsd1 RNA-Seq (Genes Up)

Functional Annotation	Genes	P-value
Topological Domain: Extracellular	60	1.2 E-07
Topological Domain: Cytoplasmic	67	2.4 E-06
Membrane Protein	101	1.7 E-05
Immunoglobulin Subtype	13	9.1 E-04
Regulation of Cell Migration	7	1.1 E-03
Rho-Gap Domain	6	1.1 E-03
GTPase Regulator	14	1.2 E-03
Cytokine Binding	7	1.2 E-03
Nucleoside Triphosphatase Regulator	14	1.3 E-03
DNA Binding Protein	11	1.8 E-03

A

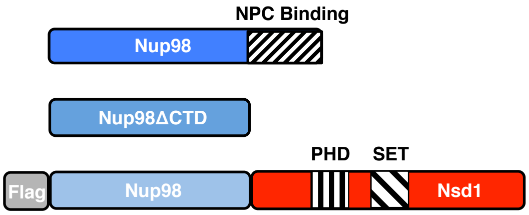

B

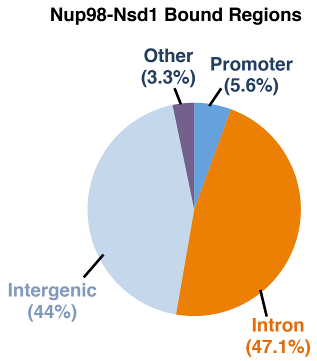

C

Functional Annotation	Genes	P-value
Regulation of Pol II Transcription	56	8 E-07
Regulation of Cell Proliferation	16	1.4 E-05
Angiogenesis	14	3.8 E-04
T-cell Differentiation	6	7.5 E-04
Ubl Conjugation	43	8.4 E-04

D

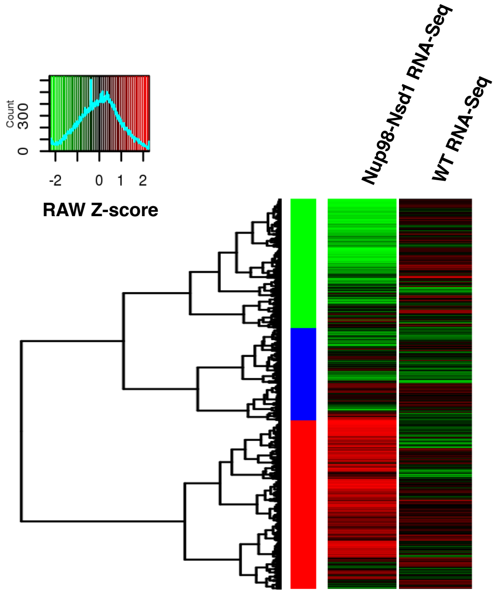

E

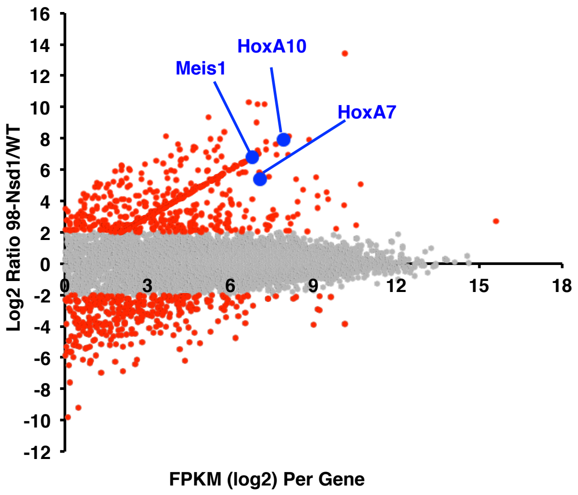

F

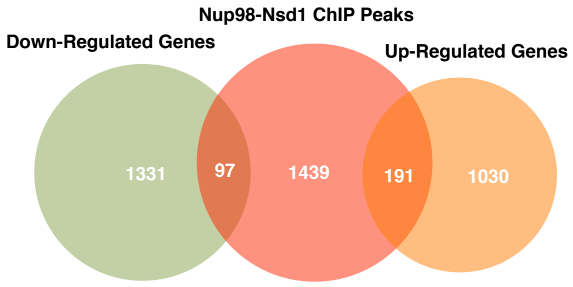

Supplement: Supplemental Material [file supp_gad.306753.117_Supplemental_Material.pdf]
